# A CMOS-Compatible Silicon Nanowire Array Natural Light Photodetector with On-Chip Temperature Compensation Using a PSO-BP Neural Network

**DOI:** 10.3390/mi17010023

**Published:** 2025-12-25

**Authors:** Mingbin Liu, Xin Chen, Jiaye Zeng, Jintao Yi, Wenhe Liu, Xinjian Qu, Junsong Zhang, Haiyan Liu, Chaoran Liu, Xun Yang, Kai Huang

**Affiliations:** 1 School of Electronic and Information Engineering, China West Normal University, Nanchong 637002, China; 2 Ministry of Education Engineering Research Center of Smart Microsensors and Microsystems, College of Electronics and Information, Hangzhou Dianzi University, Hangzhou 310018, China; 3 College of Physics and Optoelectronic Engineering, Leshan Normal University, Leshan 614000, China

**Keywords:** silicon nanowire arrays, natural light, highly controllable, low cost, PSO-BP neural network, temperature compensation

## Abstract

Silicon nanowire (SiNW) photodetectors exhibit high sensitivity for natural light detection but suffer from significant performance degradation due to thermal interference. To overcome this limitation, this paper presents a high-performance, CMOS-compatible SiNW array natural light photodetector with monolithic integration of an on-chip temperature sensor and an embedded intelligent compensation system. The device, fabricated via microfabrication techniques, features a dual-array architecture that enables simultaneous acquisition of optical and thermal signals, thereby simplifying peripheral circuitry. To achieve high-precision decoupling of the optical and thermal signals, we propose a hybrid temperature compensation algorithm that combines Particle Swarm Optimization (PSO) with a Back Propagation (BP) neural network. The PSO algorithm optimizes the initial weights and thresholds of the BP network, effectively preventing the network from getting trapped in local minima and accelerating the training process. Experimental results demonstrate that the proposed PSO-BP model achieves superior compensation accuracy and a significantly faster convergence rate compared to the traditional BP network. Furthermore, the optimized model was successfully implemented on an STM32 microcontroller. This embedded implementation validates the feasibility of real-time, high-accuracy temperature compensation, significantly enhancing the stability and reliability of the photodetector across a wide temperature range. This work provides a viable strategy for developing highly stable and integrated optical sensing systems.

## 1. Introduction

Silicon-based photodetectors are extensively employed in diverse fields, including environmental monitoring, optical communication, and biomedical imaging [1,2,3,4,5,6,7]. Silicon exhibits a broad spectral response spanning from the near-ultraviolet (NUV, ~300 nm) to the near-infrared (NIR, ~1100 nm) regions, rendering it an ideal candidate for natural light detection [8,9]. In practical applications, however, these devices frequently operate under fluctuating ambient temperatures. Due to the intrinsic semiconductor properties of silicon, the sensing elements are highly susceptible to thermal interference. This phenomenon induces a superposition of thermal and optical signals, significantly degrading detection accuracy [10]. Consequently, implementing an effective temperature compensation strategy is essential to accurately decouple the optical signal from thermal interference. This approach mitigates thermal interference in the electrical response of the sensing unit, thereby enhancing output stability and accuracy.

Silicon nanowires (SiNWs) demonstrate significantly enhanced photoresponse compared to conventional planar bulk silicon photodetectors, primarily attributed to the quantum confinement effect and efficient light-trapping properties enabled by their unique geometry. Specifically, the high surface-to-volume ratio of SiNWs induces abundant long-lived surface trap states, which prolong carrier lifetime and result in high photoconductive gain [11]. Additionally, their sub-wavelength dimensions generate strong light-trapping effects, substantially enhancing light absorption efficiency without requiring thick active layers. These factors collectively contribute to a responsivity that exceeds the theoretical limit of bulk silicon devices [12]. However, realizing these advantages in practical devices remains challenging due to fabrication limitations. For instance, Mihalach et al. [13] fabricated vertically aligned SiNW arrays via metal-assisted chemical etching (MACE). While this method offers scalability, the resulting arrays exhibited excessively high density, leading to mutual optical shadowing and a consequent sluggish response. Similarly, Zhang et al. [14] employed metal-assisted chemical vapor deposition (CVD) to fabricate large-area (>80 cm^2^) SiNW arrays. However, the synthesized SiNWs suffered from poor dimensional uniformity, resulting in significant sensor-to-sensor variation. Such inconsistencies hinder their widespread adoption in low-cost, mass-manufactured applications.

Despite continuous enhancements in material properties, thermal interference remains a persistent challenge. To address this, numerous temperature compensation strategies have been developed, primarily classified into hardware and software approaches [15,16]. Hardware compensation utilizes auxiliary circuitry to offset thermal interference in real-time; however, it is constrained by complex circuit design, lack of versatility, and limited accuracy. Conversely, software compensation, employing microprocessors for data processing, simultaneously enhances sensor linearity and temperature stability [17,18]. For instance, Shu et al. [19] improved measurement accuracy from 21.2% full scale (FS) to 0.1% FS using bilinear interpolation. Liu et al. [20] employed a Back Propagation (BP) neural network, achieving a two-order-of-magnitude improvement in both the temperature coefficient of sensitivity (TCS) and full-scale error. Similarly, Guo et al. [21] utilized Least Squares Support Vector Machines (LS-SVM) to reduce instrument temperature instability to 1.32 × 10^−3^ °/h. Li et al. [22] optimized a Kernel Extreme Learning Machine (KELM) model via the Cuckoo Search Algorithm (CSA) and Nelder–Mead algorithm, significantly enhancing compensation performance. While traditional interpolation methods are simple to implement, they often suffer from limited accuracy. Although LS-SVM exhibits excellent nonlinear modeling capabilities, it requires prolonged training time and high computational resources. Furthermore, traditional BP neural networks are prone to getting trapped in local minima and exhibit a slow convergence rate.

Various advanced techniques have been developed to address temperature-dependent interferences. For instance, multi-channel spectrapyrometry systems have successfully demonstrated precise temperature measurement without requiring prior knowledge of surface emissivity by analyzing spectral information [23]. Analogously, in the field of SiNW photodetectors, decoupling the thermal interference from the optical signal is essential. To address the thermal susceptibility of SiNW array photodetectors in variable environments, this study presents a high-performance device with integrated temperature compensation. Fabricated via standard CMOS-compatible processes, the device features high uniformity, scalability, miniaturization, and cost-effective mass production. The monolithic integration of a SiNW photodetection array and a gold-coated SiNW temperature sensor enables the simultaneous acquisition of optical and thermal signals. This architecture significantly enhances device stability while reducing peripheral circuitry complexity and power consumption. To mitigate thermal drift-induced errors, we propose a hybrid temperature compensation method combining Particle Swarm Optimization (PSO) and a Back Propagation (BP) neural network. By leveraging the global search capability of PSO to optimize the network’s initial weights and thresholds, this approach significantly improves the convergence rate. The optimized model is implemented on an STM32 microcontroller to achieve real-time compensation. Experimental results demonstrate accurate light intensity measurement under varying temperatures, validating the potential of SiNW array sensors for optical detection and environmental monitoring.

## 2. Materials and Methods

**Figure 1:**
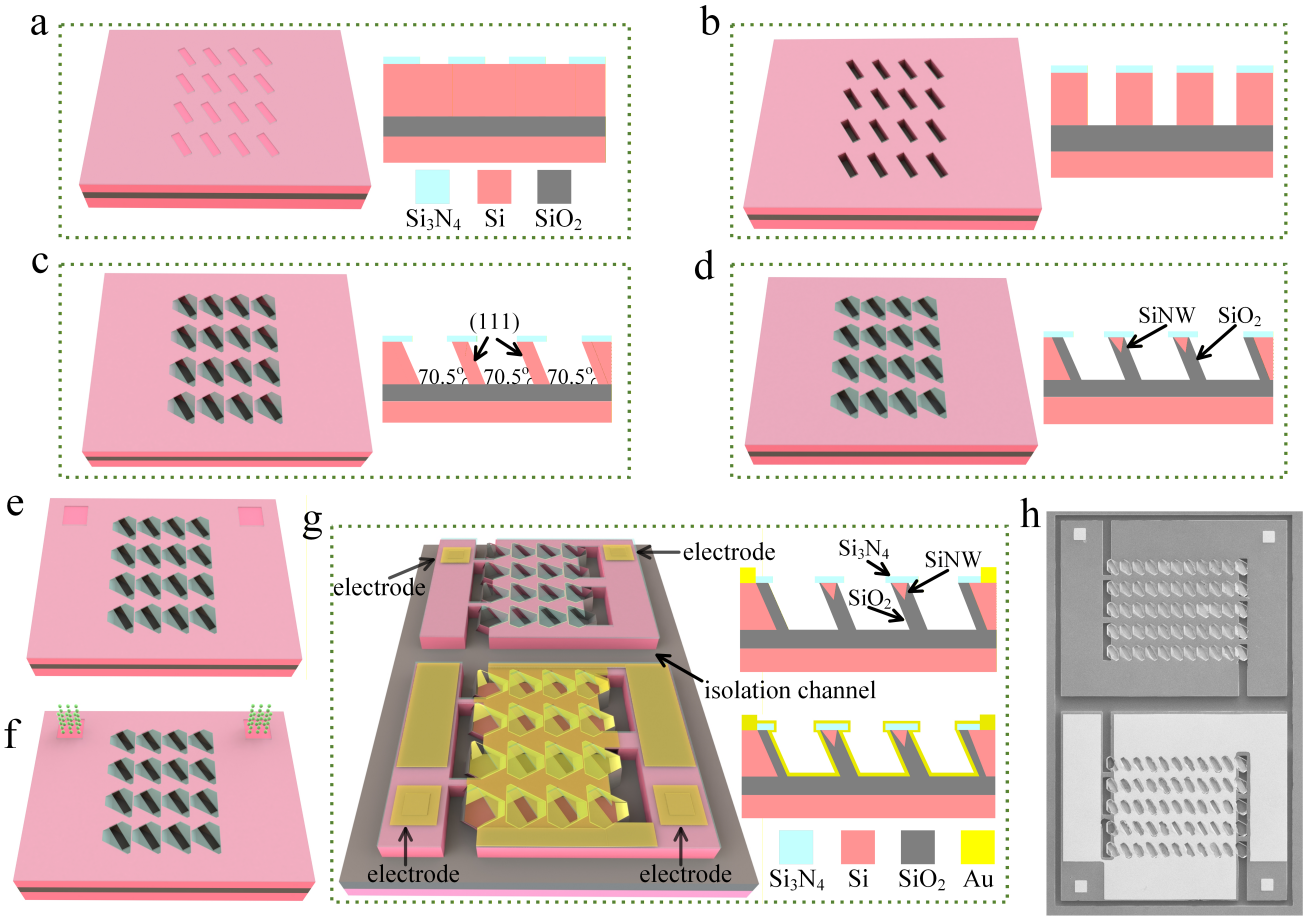
Fabrication sequence of SiNW array natural light detector. (**a**) Reactive ion etching of Si_3_N_4_ mask to define adjacent rectangular windows (cross-section shown at right); (**b**) Deep reactive-ion etching to extend the trenches to buried oxide layer; (**c**) Anisotropic wet etching in KOH solution transforming the trenches into hexagonal grooves, forming thin silicon walls bounded by (111)-oriented sidewalls; (**d**) Self-limiting oxidation forming SiNWs with triangular cross-sections at wall apex; (**e**) Reactive ion etching of silicon nitride in specific locations; (**f**) Boron ion implantation; (**g**) The fabrication of electrodes and isolation channels; (**h**) Scanning electron microscopy (SEM) image of SiNW array photodetector.

A (111)-oriented silicon-on-insulator (SOI) wafer was employed as the base substrate. The device layer of the SOI wafer was doped with boron to a concentration of 5 × 10^15^ cm^−3^. A silicon nitride (Si_3_N_4_) film was initially deposited on the device layer, followed by photolithography to pattern an array of rotated rectangular windows, as shown in Figure 1a. Subsequently, dry etching was employed to remove the Si_3_N_4_ and top silicon layer within the rectangular windows, thereby producing rectangular trenches (Figure 1b). The wafer was then immersed in a potassium hydroxide (KOH) solution for anisotropic wet etching. Owing to the much slower etch rate of the (111) crystal planes compared with other orientations, the rectangular trenches were transformed into hexagonal geometries bounded by (111) facets, forming a slanted thin-wall structure between neighboring trenches (Figure 1c) [24]. Next, the self-limiting oxidation process was conducted. As the Si_3_N_4_ atop the silicon wall retarded the oxidation process, the oxidation rate in this area was much lower than in other areas, leaving a core of unoxidized silicon, thereby forming SiNWs (Figure 1d). We use reactive ion etching technology to etch silicon nitride at specific locations to expose bulk silicon (Figure 1e), then perform boron ion implantation at that location (Figure 1f). Finally, we fabricated gold electrode at that location and etched isolation channel on the device (Figure 1g). Figure 1g presents a SiNW array natural light detector fabricated on a (111) SOI wafer. The cross-sectional diagram on the right illustrates the fully encapsulated structure of the SiNWs, which are encased by Si_3_N_4_ and silicon oxide (SiO_2_) films. The SiNW surface is chemically bonded to these insulating materials, ensuring effective surface passivation and thereby enhancing the long-term stability of the device. The ordered distribution of SiNWs on the upper surface enables rapid natural light detection, significantly improving the device’s response time. Additionally, the simultaneous operation of multiple nanowires within the array enables signal superposition, resulting in a stronger and more stable signal compared to a single SiNW device.

Furthermore, real-time monitoring of the ambient temperature is essential for implementing temperature compensation on the natural light detector. To meet this requirement, we developed an innovative dual-array structure that integrates an auxiliary gold-coated SiNW temperature array alongside the primary photodetection unit. Due to the high thermal conductivity of the gold layer, this integrated array achieves a fast thermal response and enables high-precision measurement. Figure 1h, a scanning electron microscopy (SEM) image of the detector, illustrates its layout: the upper section comprises the SiNW natural light array for optical signal acquisition; the lower section constitutes the SiNW temperature array for thermal sensing. An isolation channel physically separates the two sections, ensuring the independent operation of both arrays and preventing mutual interference. This integrated design facilitates the synchronous monitoring of optical signals and temperature variations, markedly enhancing the device’s overall performance and integration density, while significantly simplifying the peripheral circuitry.

**Figure 2:**
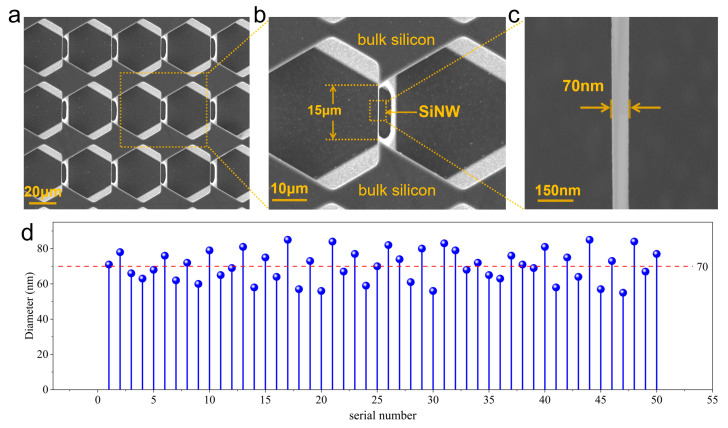
(**a**) The electron microscopy image of the completely suspended SiNW array; (**b**) the partial enlarged drawing of the SiNW array; (**c**) the electron microscopy image of SiNW; (**d**) diameter distribution of the SiNWs within the array.

Figure 2 presents scanning electron microscopy (SEM) images of the SiNW array. To obtain a fully suspended SiNW array, the Si_3_N_4_ and SiO_2_ films were removed. The ends of the SiNWs are monolithically integrated with the bulk silicon substrate, enabling reliable electrical connection without requiring complex transfer steps. Furthermore, this approach allows for the highly controllable fabrication of SiNWs with tunable lengths and diameters. As shown in Figure 2d, the SiNW diameters are centered at approximately 70 nm. In summary, through this streamlined process, we have achieved high controllability, miniaturization, and cost-effective fabrication of SiNW array devices.

## 3. Temperature Compensation Principle

**Figure 3:**
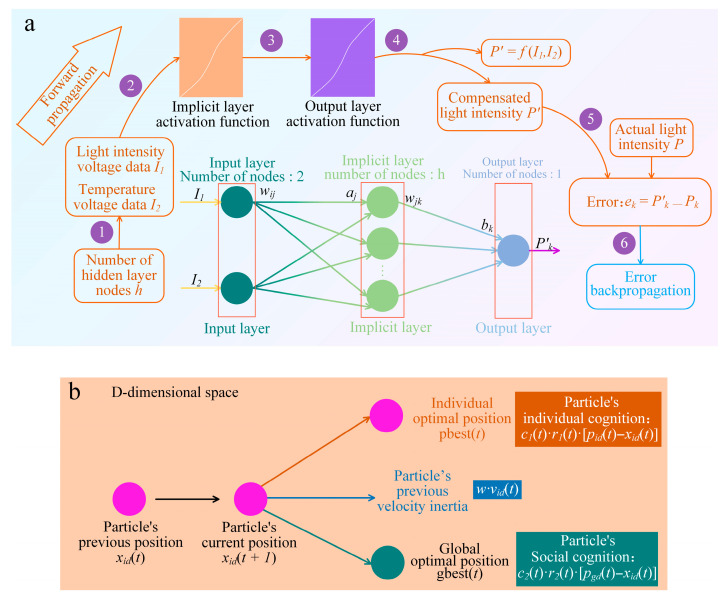
(**a**) Topology of the BP neural network for temperature compensation; (**b**) schematic of the particle velocity and position update mechanism in the PSO algorithm.

Figure 3a presents the architecture of the BP neural network applied to the temperature compensation of the natural light photodetector. This network adopts a 2–7–1 topological structure (2 input neurons, 7 hidden neurons, 1 output neuron) and consists of three layers: the input, hidden, and output layers, wherein the optimal number of hidden neurons is determined based on an empirical formula.

(1) h = (I_n_ + O_m_)^1/2^ + c

Here, c denotes a constant ranging from 1 to 10. The connection weights (w_ij_, w_jk_) and neuron thresholds are initialized; specifically, w_ij_ denotes the weight connecting the i-th neuron of the input layer to the j-th neuron of the hidden layer, while w_jk_ connects the j-th hidden neuron to the k-th output neuron. The light intensity voltage (I_1_) and temperature voltage (I_2_), acquired by the SiNW array natural light photodetector, are utilized as the network inputs. These signals propagate to the hidden layer, where nonlinear mapping is performed via an activation function to establish the relationship P’ = f(I_1_, I_2_). Subsequently, the signals reach the output layer to generate the compensated light intensity P’, representing the predicted value with temperature effects corrected. This output is compared against the standard light intensity P to calculate the error e_k_. Through the error back-propagation mechanism, the weights and thresholds are iteratively adjusted to minimize the Mean Squared Error (MSE), thereby optimizing the accuracy of the compensation model [25,26].

To improve the accuracy and convergence rate of the BP neural network for temperature compensation in the photodetector, the Particle Swarm Optimization (PSO) algorithm is employed to optimize the initial weights and thresholds. Figure 3b illustrates the position update mechanism of particles within the search space. PSO is a stochastic optimization method based on swarm intelligence, where multiple particles collaboratively search the solution space to iteratively approach the global optimum [27,28]. The update mechanism is governed by three components: the cognitive component (representing individual learning from the personal best, p_best_), the social component (representing collective learning from the global best, g_best_), and the inertia component (based on the previous velocity, w·v_id_), which balances the trade-off between global exploration and local exploitation. In each iteration, particles update their velocities and positions guided by both individual experience and social interaction, thereby converging towards the global optimum. The mathematical model of the update process is described by the following equations:

(2) v_id_(t + 1) = w × v_id_(t) + c_1_ × r_1_(t) × [p_id_(t) − x_id_(t)] + c_2_ × r_2_(t) × [p_gd_(t) − x_id_(t)]

(3) x_id_(t + 1) = x_id_(t) + v_id_(t + 1)

In these equations, w represents the inertia weight; a larger w facilitates global exploration, while a smaller value favors local exploitation. c_1_ and c_2_ denote the learning factors (acceleration constants), commonly set to 2, whereas r_1_ and r_2_ represent random numbers uniformly distributed in the interval [0, 1]. Additionally, t denotes the current iteration, while v_id_(t) and x_id_(t) represent the velocity and position of the i-th particle in the d-th dimension at iteration t, respectively. By utilizing this mechanism, PSO effectively identifies optimal initial parameters, preventing the traditional BP network from getting trapped in local minima caused by sensitivity to initial weights, thereby enhancing the stability and prediction accuracy of the temperature compensation model.

## 4. Results and Discussion

### 4.1. Construction of the Testing System

**Figure 4:**
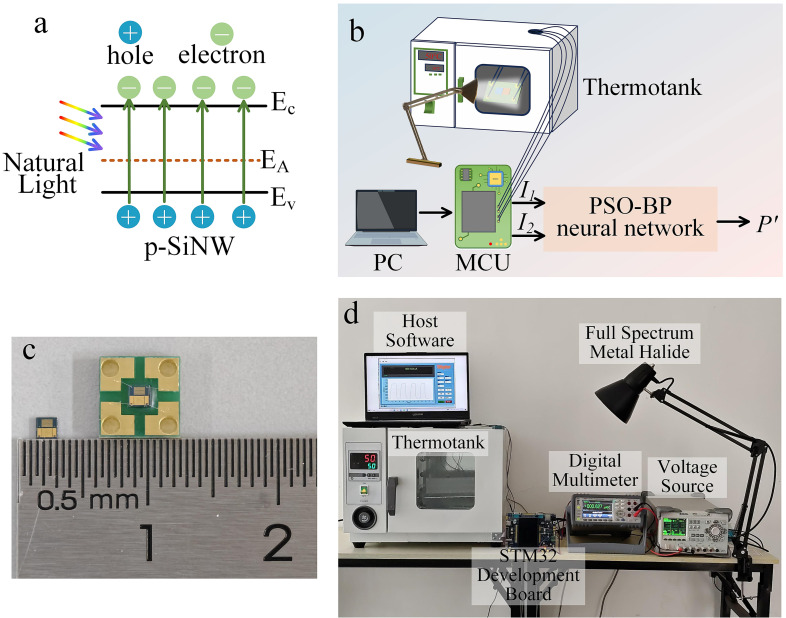
(**a**) The sensing mechanism; (**b**) schematic diagram of the temperature compensation test for the natural light detector; (**c**) the SiNW natural light detector chip and the encapsulated device; (**d**) experimental setup for temperature compensation.

Figure 4c displays the photographs of the fabricated SiNW photodetector chip and its encapsulated device, while Figure 4d depicts the experimental setup employed for temperature compensation. As shown in Figure 4d, the device is powered by a RIGOL DP832 DC power supply, with electrical signals acquired by a Tonghui TH1963 digital multimeter. Data are transmitted in real-time and stored on a computer for analysis. Additionally, a full-spectrum metal halide lamp provides illumination, a thermotank regulates the ambient temperature, and an STM32 microcontroller collects the output voltage signal. The sensing mechanism is based on the photoconductive effect. Under illumination, the photoresponse arises primarily from intrinsic absorption where valence band electrons are excited to the conduction band. Simultaneously, the high surface-to-volume ratio of SiNWs introduces a high density of localized surface states [29]. These states capture photogenerated carriers, inhibiting immediate recombination and prolonging the carrier lifetime, thereby modulating the channel conductivity. In practical operation, both light intensity and ambient temperature simultaneously affect the detector’s response, introducing thermal interference into the output signal. To obtain accurate light intensity, a temperature compensation strategy is implemented. The voltage signals corresponding to light intensity (I_1_) and temperature (I_2_), acquired by a microcontroller, serve as inputs to the neural network. The network processes these inputs to generate a compensated light intensity (P’), which approximates the standard light intensity (P) by minimizing temperature-induced errors.

### 4.2. Response Test

**Figure 5:**
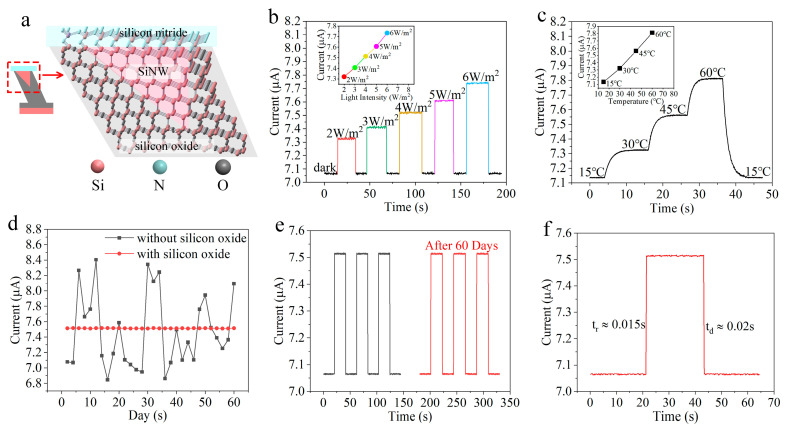
(**a**) Schematic of the passivated SiNWs structure; (**b**) time-dependent photo-response of the detector; (**c**) current variation trends of the SiNW temperature sensing array within the temperature range of 15–60 °C; (**d**) comparison of long-term stability between the encapsulated and bare detectors; (**e**) comparison of current measurement results before and after a 60-day storage period; (**f**) magnified view of the photocurrent signal.

As illustrated in Figure 5a, a dual-layer passivation structure was constructed by encapsulating the SiNW array with Si_3_N_4_ and SiO_2_ thin films, thereby establishing a protective barrier with excellent chemical stability and electrical insulation. This configuration effectively isolates the device from the ambient environment, mitigating surface degradation and the resulting drift in electrical properties, thereby significantly enhancing long-term stability and signal consistency. The optoelectronic performance of the detector was characterized at an ambient temperature of 22 °C. We used a full-spectrum metal halide lamp to simulate natural light illumination. The optical power density was calibrated using an optical power meter to ensure measurement accuracy. Under a bias of +0.5 V, the optical power density was incrementally increased from dark conditions to the range of 2–6 W·m^−2^, as depicted in Figure 5b. With increasing optical power, electrons in the valence band are excited, generating a higher concentration of carriers via band-to-band transition. This leads to a progressive increase in photocurrent, demonstrating excellent responsivity. Furthermore, the thermal response of the integrated temperature sensing array was examined under varying thermal environments. Regarding the intrinsic characteristics of the sensor element, the gold-coated SiNW temperature sensor exhibits a resistance sensitivity of 237.47 Ω/°C. This intrinsic property and its detailed characterization were reported in our previous work [30]. During the experiment, the device was subjected to controlled temperatures ranging from 15 °C to 60 °C in increments of 15 °C to assess performance stability. Figure 5c illustrates the variation of current signals versus temperature variations. As observed, the current signal increases stepwise with rising temperature and achieves thermal equilibrium within approximately 6 s, exhibiting a rapid response. Given the high surface-to-volume ratio of SiNWs, they are susceptible to surface oxidation and adsorption, causing temporal resistance fluctuations that compromise stability. Figure 5d compares the performance of the fully encapsulated SiNW detector with a bare SiNW counterpart. Figure 5e further illustrates the performance comparison before and after a 60-day aging period. Experimental data demonstrate that the proposed passivation significantly enhances reliability. Figure 5f presents an enlarged view of the photo-response signal is presented to facilitate the analysis of parameters such as response time, power consumption, signal amplitude, and signal-to-noise ratio (SNR). Under 4 W·m^−2^ illumination, the detector achieves a signal of 0.51 μA with a power consumption of 3.75 μW. Additionally, the uncorrelated noise from individual SiNWs sums incoherently. Consequently, the SiNW array detector exhibits a high SNR, attributed to the noise averaging effect inherent to the array architecture.

(4) SNR = 20 × lg(A_signal_/A_noise_)

where A_signal_ and A_noise_ denote the signal amplitude and noise amplitude, respectively. The signal-to-noise ratio (SNR) of the detector was calculated to be 65.4 dB.

Due to the intrinsic semiconducting properties of silicon, the SiNW sensing element of the photodetector is sensitive to ambient temperature, which induces significant thermal interference. This interference results in the coupling of temperature and optical signals within the output, thereby degrading the measurement accuracy. To mitigate this issue, a systematic temperature compensation experiment was performed. The experiment spanned the temperature range of 10–60 °C, with 11 test points set at intervals of 5 °C. At each temperature, 11 optical power density levels (ranging from 2 to 12 W·m^−2^ with a step size of 1 W·m^−2^) were applied to construct a comprehensive dataset characterizing the coupled thermo-optical response.

**Figure 6:**
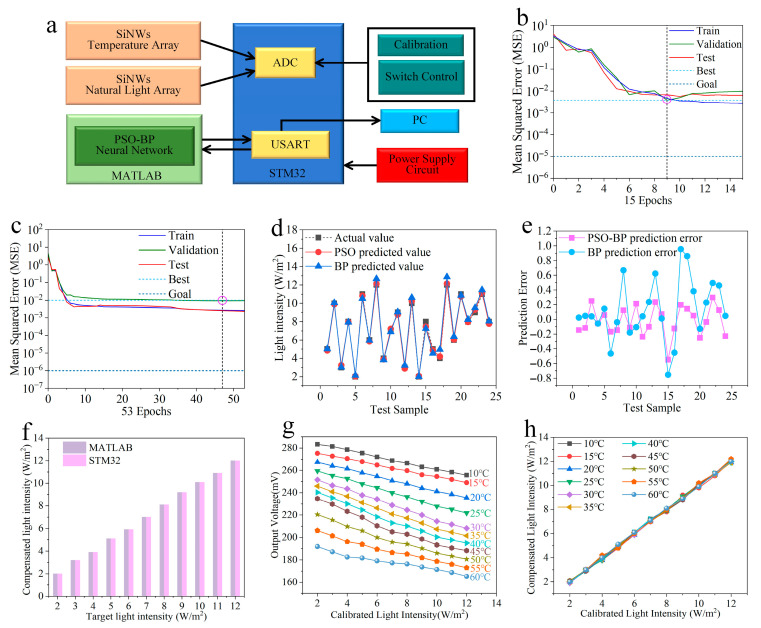
(**a**) Schematic of the temperature compensation experimental setup; (**b**) training performance curves of the PSO-BP neural network; (**c**) training performance curves of the traditional BP neural network; (**d**) comparison of prediction results on the test set between the traditional BP and PSO-BP models; (**e**) comparison of relative errors of the two models; (**f**) comparison of compensation results between MATLAB simulation and STM32 hardware implementation under varying optical power densities at 30 °C; (**g**) temperature-dependent response characteristics of the SiNW array photodetector; (**h**) compensated optical power density outputs processed by the PSO-BP neural network.

Figure 6a illustrates the schematic of the temperature compensation experiment. Due to the high signal amplitude and stability of the SiNW array sensor, the readout circuit is significantly simplified. Specifically, the output voltage signals (I_1_ and I_2_) from the SiNW photodetector were directly acquired via the integrated analog-to-digital converter (ADC) of an STM32 microcontroller.

During the experiment, the SiNW array photodetector integrated with a temperature sensor (as shown in Figure 1), was placed within a high-precision temperature-controlled chamber. Specifically, the experiment was conducted by stabilizing the temperature at 10 °C while incrementally adjusting the optical power density from 2 to 12 W·m^−2^. Upon reaching thermal equilibrium, the corresponding output voltages were recorded. Subsequently, the temperature was adjusted to the next level (step size: 5 °C), and the optical sweep was repeated until the entire dataset was acquired. The calibration results of the SiNW array photodetector are presented in Figure 6g. The figure reveals distinct output variations under identical illumination but varying temperatures, confirming the significant impact of thermal interference. The collected data were transmitted from the STM32 to MATLAB 2020 via the USART protocol for subsequent processing. A total of 121 sample sets were collected; 97 samples were randomly selected as the training set, while the remaining 24 served as the test set. Next, the PSO-BP model was constructed with an architecture of 2 input neurons and 1 output neuron, with the number of optimal hidden neurons determined via an empirical formula. The hidden and output layers adopted the hyperbolic tangent sigmoid (tansig) and linear (purelin) transfer functions, respectively. The hyperparameters were configured as follows: maximum epochs = 1000, target error = 1 × 10^−5^, training function = trainlm, learning rate = 0.01, population size = 30, inertia weight w = 0.9, and learning factors c_1_ = 2, c_2_ = 2. The training performance curves of the PSO-BP and traditional BP models are illustrated in Figure 6b and Figure 6c, respectively. The PSO-BP model converged in 15 epochs, achieving a minimum Mean Squared Error (MSE) of 0.0037799 at epoch 9; in contrast, the traditional BP model required 53 epochs to reach a minimum MSE of 0.0092937 at epoch 47. This indicates that the optimized network exhibits a superior convergence rate by requiring fewer epochs, and effectively avoids local minima as evidenced by the lower MSE. To evaluate model generalization, the prediction accuracy on the test set was compared, as shown in Figure 6d,e. It is evident that the predicted values from the PSO-BP model exhibit higher consistency with the standard values, demonstrating the robustness and efficacy of the optimization algorithm.

Subsequently, the experimental data were processed by the trained PSO-BP model to generate temperature-compensated optical power density predictions, as depicted in Figure 6h. Analysis of the relative errors indicates that the proposed compensation method reduced the temperature-induced error from approximately 14% to 1.42%, demonstrating a significant improvement in measurement accuracy. It can be observed that the compensated values exhibit high consistency with the standard values across the entire temperature range. These results confirm that the optimized network effectively mitigates temperature-induced errors, thereby demonstrating superior accuracy and stability.

Following training, the PSO-BP model was deployed from MATLAB to an STM32 microcontroller. To ensure computational precision during embedded execution, the STM32F4 series microcontroller was selected as the hardware platform. First, the optimal weights and thresholds were extracted from the converged PSO-BP model and mapped into C arrays within the Keil development environment. Since the microcontroller operates independently of MATLAB, the network’s forward propagation algorithm was implemented in C code, strictly following the architecture and computational mechanisms of the BP neural network. The computation entails calculating hidden layer inputs, generating outputs via the hyperbolic tangent sigmoid (tansig) function, determining output layer inputs, and deriving the final result using the linear (purelin) function. These routines were encapsulated as modular functions within the Keil environment to facilitate efficient invocation during the inference phase. Following implementation, the temperature compensation algorithm was executed and validated on the STM32 microcontroller, adhering to the operational principles outlined in Figure 6a. Given the requirement for high-precision numerical computation, the standard math library was utilized to ensure calculation accuracy and real-time performance. As illustrated in Figure 6f, the PSO-BP model was simulated in MATLAB and executed on the STM32 platform to perform temperature compensation at a test temperature of 30 °C. The network inference yielded compensated optical power density values, effectively mitigating thermal interference. The observed consistency between the simulation (MATLAB) and hardware (STM32) results confirms the successful deployment of the model, validating the feasibility of embedded temperature compensation.

**Figure 7:**
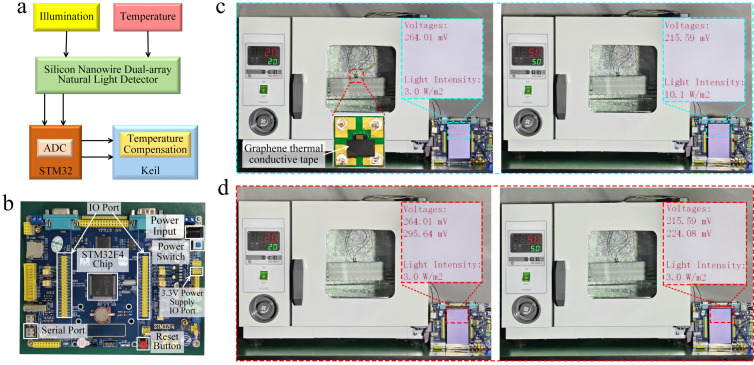
(**a**) Flowchart of the embedded temperature compensation algorithm; (**b**) photograph of the STM32F4 hardware platform; (**c**) uncompensated optical power density measurements of the SiNW photodetector at 20 °C and 50 °C; (**d**) compensated optical power density measurements at 20 °C and 50 °C.

Upon the deployment of the neural network model on the STM32F4 microcontroller, experimental validation of temperature compensation for the SiNW array photodetector was performed. Figure 7a depicts the flowchart of the STM32F4-based temperature compensation procedure, while Figure 7b displays the photograph of the STM32F4 development board. The system is based on a dual-array SiNW photodetector, which simultaneously captures optical and temperature signals, converting them into analog voltage signals. Following acquisition via the integrated ADC of the STM32, the dual-channel data are processed by the embedded PSO-BP algorithm, thereby enabling real-time temperature compensation of the optical signal. To validate the compensation efficacy, the device was subjected to an optical power density of 3 W/m^2^ under two distinct thermal environments: 20 °C and 50 °C. To mitigate optical interference on the temperature array, opaque graphene tape was employed to optically isolate the sensing area prior to testing, ensuring measurement accuracy. Figure 7c shows the output characteristics prior to compensation. Although the optical power density was maintained at 3 W/m^2^, the coupling of thermal interference into the output signal resulted in significant deviations in measured values. Figure 7d displays the results obtained after applying temperature compensation. The compensation algorithm effectively mitigated thermal effects, resulting in highly accurate optical power density measurements.

## 5. Conclusions

This work proposes a temperature compensation strategy utilizing a Particle Swarm Optimization (PSO)-enhanced Back Propagation (BP) neural network. Experimental validation confirms that the proposed method significantly improves compensation accuracy and system stability under varying thermal environments. To enhance thermal sensitivity, a gold-coated SiNW array structure was integrated into the photodetector. Leveraging the high thermal conductivity of gold, this design facilitates a rapid thermal response to ambient fluctuations, thereby enhancing the sensitivity and accuracy of temperature monitoring. A dual-array architecture was developed, comprising a photosensitive SiNW array and a thermosensitive gold-coated SiNW array. This configuration enables the simultaneous acquisition of optical and temperature signals, which improves sensor integration and versatility while simplifying the peripheral circuitry, thereby reducing power consumption and system complexity. Furthermore, a dual-layer passivation structure was developed to protect the SiNWs, significantly improving the detector’s long-term stability and reliability. In terms of algorithm optimization, PSO was employed to globally optimize the initial weights and thresholds of the BP neural network. This approach mitigates inherent BP limitations, specifically slow convergence rate and the tendency of getting trapped in local minima. Experimental results indicate that the PSO-BP model exhibits superior compensation accuracy and convergence rate. It reduces training time and avoids entrapment in local minima associated with traditional BP networks. To enhance integration, the pre-trained neural network was deployed onto an STM32 microcontroller, realizing temperature compensation via an embedded implementation. This strategy significantly improves the compactness and autonomy of the system. Validation confirms that the neural network maintains high precision when deployed on the STM32 platform. It effectively mitigates thermal interference caused by real-time temperature fluctuations, enhancing sensor stability and precision. These findings provide a significant technical foundation for expanding the application of SiNW array sensors in optical detection.

## Data Availability

The original contributions presented in the study are included in the article, further inquiries can be directed to the corresponding author.

## References

[1] De Vita C., Toso F., Pruiti N.G., Klitis C., Ferrari G., Sorel M., Melloni A., Morichetti F. (2022). Amorphous-silicon visible-light detector integrated on silicon nitride waveguides. Opt. Lett..

[2] Huang Q., Tao L., Zhu H., Lin W., Chen J., Fang Y. (2025). Silicon-based narrowband photodetectors with blade-coated perovskite light extinction layer for high performance visible-blind NIR detection. J. Mater. Sci. Mater. Electron..

[3] Zhang M., Jia Z., Lv X., Huang X. (2020). Biological detection based on the transmitted light image from a porous silicon microcavity. IEEE Sens. J..

[4] Yang J., Jia Z., Lü X., Huang X., Wang J. (2021). Digital image biological detection technology based on the porous silicon periodic crystals film. Optoelectron. Lett..

[5] Lin Y., Yong Z., Luo X., Azadeh S.S., Mikkelsen J.C., Sharma A., Chen H., Mak J.C.C., Lo P.G.-Q., Sacher W.D. (2022). Monolithically integrated, broadband, high-efficiency silicon nitride-on-silicon waveguide photodetectors in a visible-light integrated photonics platform. Nat. Commun..

[6] Cai S., Xu X., Yang W., Chen J., Fang X. (2019). Materials and designs for wearable photodetectors. Adv. Mater..

[7] Tsai M.L., Tsai D.S., Tang L., Chen L.J., Lau S.P., He J.H. (2017). Omnidirectional harvesting of weak light using a graphene quantum dot-modified organic/silicon hybrid device. ACS Nano.

[8] Fang Z., Zhao C.Z. (2012). Recent progress in silicon photonics: A review. Int. Sch. Res. Not..

[9] Liu C., Guo J., Yu L., Li J., Zhang M., Li H., Shi Y., Dai D. (2021). Silicon/2D-material photodetectors: From near-infrared to mid-infrared. Light Sci. Appl..

[10] Norton P., Brandt J. (1978). Temperature coefficient of resistance for p-and n-type silicon. Solid-State Electron..

[11] Zhang A., You S., Soci C., Liu Y., Wang D., Lo Y.-H. (2008). Silicon nanowire detectors showing phototransistive gain. Appl. Phys. Lett..

[12] Liang M.F., Fu C., Li Y.H., Yu B., Shi F., Gao Y.M., Long S.J., Liang F.X., Luo L.B. (2025). Study of Ultranarrowband Silicon Nanowire Array Photodetector with Peak Spectral Responsivity at 1120 nm. ACS Nano.

[13] Mihalache I., Radoi A., Pascu R., Romanitan C., Vasile E., Kusko M. (2017). Engineering graphene quantum dots for enhanced ultraviolet and visible light p-Si nanowire-based photodetector. ACS Appl. Mater. Interfaces.

[14] Zhang B.C., Jie J.S., Shao Z.B., Huang S.Y., He L., Zhang X.H. (2019). One-step growth of large-area silicon nanowire fabrics for high-performance multifunctional wearable sensors. Nano Res..

[15] Ramírez-Muñoz D., García-Gil R., Cardoso S., Freitas P. (2024). Characterization of Magnetoresistive Shunts and Its Sensitivity Temperature Compensation. Sensors.

[16] Li J., Pan F., Li J., Ji Y., Song H., Wang B. (2023). Research on TMR current transducer with temperature compensation based on reference magnetic field. IEEE Access.

[17] Wu J., Zhou K., Jin Q., Lu B., Jin Z., Chen J. (2024). A High-Precision Temperature Compensation Method for TMR Weak Current Sensors Based on FPGA. Micromachines.

[18] Včelák J., Ripka P., Platil A., Kubík J., Kašpar P. (2006). Errors of AMR compass and methods of their compensation. Sens. Actuators A Phys..

[19] Shu Y., Hua C., Zhao Z., Wang P., Zhang H., Yu W., Yu H. (2024). Temperature Compensation Method Based on Bilinear Interpolation for Downhole High-Temperature Pressure Sensors. Sensors.

[20] Liu H., Li H.J. (2020). Research on Temperature Compensation Method of Pressure Sensor Based on BP Neural Network. Chin. J. Sens. Actuators.

[21] Wei G., Li G., Wu Y., Long X. (2011). Application of Least Squares-Support Vector Machine in system-level temperature compensation of ring laser gyroscope. Measurement.

[22] Li J., Hu G., Zhou Y., Zou C., Peng W., Alam Sm J. (2017). Study on temperature and synthetic compensation of piezo-resistive differential pressure sensors by coupled simulated annealing and simplex optimized kernel extreme learning machine. Sensors.

[23] Magunov A.N., Pylnev M.A., Lapshinov B.A. (2014). Spectral pyrometry of objects with unknown emissivities in a temperature range of 400–1200 K. Instrum. Exp. Tech..

[24] Oosterbroek R., Berenschot J., Jansen H., Nijdam A., Pandraud G., Berg A.v.D., Elwenspoek M. (2000). Etching methodologies in〈111〉-oriented silicon wafers. J. Microelectromech. Syst..

[25] Buscema M. (1998). Back propagation neural networks. Subst. Use Misuse.

[26] Li J., Cheng J.-H., Shi J.-Y., Huang F. (2012). Brief introduction of back propagation (BP) neural network algorithm and its improvement. Advances in Computer Science and Information Engineering: Volume 2.

[27] Li Y., Li Y., Li F., Zhao B., Li Q. (2015). The Research of Temperature Compensation for Thermopile Sensor Based on Improved PSO-BP Algorithm. Math. Probl. Eng..

[28] Wu W., Yao B., Huang J., Sun S., Zhang F., He Z., Tang T., Gao R. (2023). Optimal temperature and humidity control for autonomous control system based on PSO-BP neural networks. IET Control Theory Appl..

[29] Wang H., Wang F., Xu T., Xia H., Xie R., Zhou X., Ge X., Liu W., Zhu Y., Sun L. (2021). Slowing hot-electron relaxation in mix-phase nanowires for hot-carrier photovoltaics. Nano Lett..

[30] Chen X., Zeng J., Liu M., Liu J., Zheng C., Yi J., Liu W., Qu X., Yang X., Liu W. (2025). High-Performance Silicon Nanowire Array Electronic Thermometer Fabricated Using CMOS-MEMS Techniques. ACS Appl. Electron. Mater..

